# No evidence of binding items to spatial configuration representations in visual working memory

**DOI:** 10.3758/s13421-018-0814-8

**Published:** 2018-05-17

**Authors:** Rob Udale, Simon Farrell, Christopher Kent

**Affiliations:** 1 School of Experimental Psychology, University of Bristol, Bristol, UK; 2 University of Western Australia, Crawley, Australia; 3 University of Bristol, Bristol, UK

**Keywords:** Visual working memory, Change detection, Feature binding, Location binding, Relational encoding

## Abstract

When detecting changes in visual features (e.g., colour or shape), object locations, represented as points within a configuration, might also be automatically represented in working memory. If the configuration of a scene is represented automatically, the locations of individual items might form part of this representation, irrespective of their relevance to the task. Participants took part in a change-detection task in which they studied displays containing different sets of items (shapes, letters, objects), which varied in their task relevance. Specifically, they were asked to remember the features of two sets, and ignore the third set. During the retention interval, an audio cue indicated which of the to-be-remembered sets would become the target set (having a 50% probability of containing a new feature). At test, they were asked to indicate whether a new feature was present amongst the target set. We measured binding of individual items to the configuration by manipulating the locations of the different sets so that their position in the test display either matched or mismatched their original location in the study display. If items are automatically bound to the configuration, location changes should disrupt performance, even if they were explicitly instructed not to remember the features of that particular set of items. There was no effect on performance of changing the locations of any of the sets between study and test displays, indicating that the configural representation did not enter their decision stage, and therefore that individual item representations are not necessarily bound to the configuration.

In this article, we examine the role of spatial configurations in visual working memory (VWM). Spatial configurations are important for visual cognition (Chun, 2000) and maintaining visual features in memory (Jiang, Olson, & Chun, 2000). For example, imagine that you are sitting at your desk, drinking a cup of coffee. You put your mug down momentarily to use your computer. In order to pick your mug back up, you need to be able to temporarily maintain what the mug looked like (its colour, shape, size, etc.), and its location on the desk, relative to the other items on the desk. This ability allows you to accurately pick up and drink from the mug, rather than the mouse that happened to be nearby on the desk. The core question addressed here is how spatial configurations might underpin how we organise scene representations (Hollingworth, 2006, 2007; Inoue & Takeda, 2012).

A number of existing theories on VWM assume that encoding the spatial configuration of items in a scene (e.g., the items on the desk) is necessary for remembering the visual features (e.g., colour and shape) of those items (Blalock & Clegg, 2010; Boduroglu & Shah, 2006; Jiang et al., 2000; Lin & He, 2012; Mou, Xiao, & McNamara, 2008; Treisman & Zhang, 2006). For example, the structural gist theory (Vidal, Gauchou, Tallon-Baudry, & O’Regan, 2005) states that when multiple items are maintained in VWM, a representation called the *structural gist* is formed, which contains information about the entire set of items and the relations between them. This representation serves as a web of interitem relations, and is reinstated in order to retrieve visual features about individual items (Vidal et al., 2005). Furthermore, Jiang et al. (2000) and others (Blalock & Clegg, 2010; Boduroglu & Shah, 2006; Lin & He, 2012; Mou et al., 2008; Phillips, 1974; Woodman, Vogel, & Luck, 2012) have presented evidence that the spatial configuration of a display of to-be-remembered items is maintained in memory and used to access visual features during the decision stages of a change-detection task. Jiang et al. (2000) asked participants to detect changes in displays of coloured items and to ignore changes in their locations. Colour change-detection performance was made worse when the test display items were presented in new locations, such that the spatial configuration of the test display did not match the spatial configuration of the study display. However, performance was unaffected when the locations of the items changed in such a way so as to preserve the original configuration, for example, by expanding their locations away from the centre of the display. This led to the conclusion that individual items in a display are not represented independently but contain additional information about their relative (invariant to distance) positions to one another.

One question that remains unanswered is the extent to which we have control over when and how we use this relational information. Specifically, are relational representations *top-down dependent*, in that relational representations only contain information about task relevant items (Hollingworth, 2006; Jiang et al., 2000)? Or, are they *top-down independent* (Inoue & Takeda, 2012), in that the relational representations contain information about all of the items in a display, irrespective of the task relevance of individual items? These questions relate to a debate on the extent to which we have control over the contents of our memory (Udale, Farrell, & Kent, 2017a, b). On the one hand, it is thought that we have a high degree of volitional control over the contents of memory. For example, when an item is retroactively cued after the offset of a study display, observers are more accurate at retrieving the features of that item (Gazzaley & Nobre, 2012; Lepsien, Griffin, Devlin, & Nobre, 2005; Makovski, Sussman, & Jiang, 2008; Lepsien & Nobre, 2005; Nobre et al., 2004; Nobre, Griffin, & Rao, 2007), suggesting that they are able to reallocate memory resources from task-irrelevant items to task-relevant items. Furthermore, in the context of verbal short-term memory, it is thought that removal of outdated information is a fundamental and active process (Ecker, Lewandowsky, & Oberauer, 2014; Ecker, Lewandowsky, Oberauer, & Chee, 2010; Lendínez, Pelegrina, & Lechuga, 2011; Miyake et al., 2000). On the other hand, there is a view that we have poor volitional control over the contents of memory, such that irrelevant items often enter memory, and that we frequently fail to remove irrelevant distractors from VWM, particularly when encoding times are short (cf. Olson, Moore, & Drowos, 2008, Experiment 5). Olson et al. (2008) presented study displays of shapes or faces, some of which were cued. Participants were instructed to encode only the cued items. At test, participants where shown one of the cued items (in which case, they should respond ‘same’); an uncued distractor (in which case, they should respond ‘different’); or a never-before-seen lure (and respond ‘different’). Participants had a greater bias towards responding ‘same’ when the distractors (which were presented in the study display, but were uncued) were presented at test, than when never-before seen lures were presented at test. We interpret this result as indicating that the distractors were necessarily encoded to memory and suggest that this shows we have poor top-down control over the contents of memory.

If observers have poor control over the encoding of visual features (Olson et al., 2008), and the spatial configurations of a scene may be important for maintaining and retrieving visual features (Jiang et al., 2000; Treisman & Zhang, 2006), then to what extent do we have control over the encoding of a display’s spatial configuration? The structural gist theory, among others, suggests that the spatial relationships between items are encoded in a relatively automatic manner, independent of top-down attention (Biederman, 1972; Li, VanRullen, Koch, & Perona, 2002; Vidal et al., 2005; Yang, Tsen, & Wu, 2015). If the configuration of a scene is encoded automatically, we expect the configural representation should be composed of all of the items in a display, irrespective of whether or not the observers were explicitly instructed to encode specific individual items within the display. When instructed to remember only a subset of items in a display (e.g., Olson et al., 2008), will the configural representation constitute only those task-relevant items, or will it include all of the items, irrespective of their task-relevance?

Previous research has investigated the extent to which parts of the spatial configuration enter VWM (Hollingworth, 2006, 2007; Inoue & Takeda, 2012; Sun & Gordon, 2010). For example, Inoue and Takeda (2012) asked participants to study displays containing pictures of scenes, which were segmented by horizontal and vertical lines, forming boxes. Some of the boxes were cued prior to the study display, and the participants were instructed to remember the features within the cued boxes. Swapping the contents of the cued boxes between study and test made performance worse, whereas swapping the uncued boxes did not. One explanation for this finding is that the participants were utilising the configuration to access the features of the task-relevant boxes (Inoue & Takeda, 2012). However, it is not possible to ascertain from their design whether the locations of the task-relevant boxes were incorporated into the configuration, because the boxes only ever swapped locations, and were not presented in completely new, previously unoccupied locations, so that the overall spatial configuration (four quadrants) was disrupted. Presenting to-be-ignored items in completely new locations may make it easier to detect whether the locations of those items form part of the configuration or affect the ability to rely on the encoded spatial configuration at test.

Furthermore, Jiang et al. (2000) found no effect of changing the locations of task-irrelevant items. However, it is possible that the task-irrelevant items were filtered out prior to encoding in their experiment: All of the task-irrelevant items shared the same colour, and so perceptual grouping may have allowed items to be encoded or ignored en masse. Additionally, Sun and Gordon (2010) found that changing the locations of contextual objects (i.e., objects presented during study and test displays, but which were not cued for retrieval) made performance worse for the target objects, suggesting that the locations of the contextual items formed part of the configuration. Further, their results indicate that this configural encoding may have occurred irrespective of top-down selective attention. Sun and Gordon made changes to the colour, orientations, or locations of contextual items (items present in the study and probe displays, but which were not probed themselves). Changing the colours or orientations of the contextual items made performance worse when participants were tasked with detecting changes in colour or orientation, respectively. In contrast, changes in the locations of the context items had a consistent detrimental effect, despite location never being a task-relevant feature. Our interpretation of these findings is that contextual encoding of colour or orientation is susceptible to selective-attention, whereas contextual encoding of location is not.

Finally, in contrast to the studies described above, in a colour change detection task, Woodman et al. (2012) found that changing the (task-irrelevant) configuration of the display by changing the location of colours—including scrambling their locations—had no effect on whole-probe performance. However, when a single item was spatially cued during the probe display, changes in location did disrupt performance. One possible explanation for these results is that when probed items are cued spatially, participants might strategically encode the configuration in order to make use of the spatial information on the unscrambled trials (Woodman et al., 2012). This interpretation suggests the possibility that the encoding of the display’s configuration might be subject to goal-directed top-down attention.

To summarise, whether visual features are encoded (and subsequently maintained and retrieved) appears to be determined by their relevance to the task (e.g., Sun & Gordon, 2010). However, to what extent is the encoding and maintenance of spatial configuration also influenced by goal-directed top-down selection? According to top-down independent theories (e.g., Sun & Gordon, 2010; Vidal et al., 2005), the spatial locations of all of the items in a scene should form part of the scene’s spatial representation, irrespective of the task relevance of the individual items. However, if we have control over which items form part of the configuration, it may be possible to prevent the encoding of task-irrelevant item locations and to remove the locations of items which become task-irrelevant during the maintenance interval. One issue with many studies in the literature is that all locations are potentially relevant during encoding, as the participants do not know which items may subsequently become irrelevant. For example, in the study by Sun and Gordon (2010), the target item is not identified until the probe display. This common feature of experiments was part of our motivation for these experiments—Will the locations of items which people know will never be probed form part of the configuration in memory? If not, this would provide evidence against the notion that locations are obligatorily encoded.

A related question is whether attention influences the encoding of a configuration. We were interested in whether attention can influence the spatial representation after it has been encoded. A large body of evidence using the retro-cue paradigm shows that observers have some control over whether visual features are encoded or removed from memory, and therefore the extent to which those features enter the decision stage (Gazzaley & Nobre, 2012; Lepsein, Griffin, Devlin, & Nobre, 2005; Makovski, Sussman, & Jiang, 2008; Nobre et al., 2004; Nobre et al., 2007). Thus, we set out to investigate the extent to which selective attention can modulate which items form part of the spatial configuration representation, either by adding them during encoding or removing them during maintenance. In the experiments reported here, we test the role of attention in spatial representations by manipulating the task-relevance of subsets of items in a display of to-be-remembered items. Participants were instructed to encode two sets of stimuli and ignore a third set. During maintenance, they were cued as to the set which would subsequently be probed—encouraging them to drop the other set from memory (Maxcey & Woodman, 2014; Williams, Hon, Kang, Carlisle, & Woodman, 2013). Depending on how selective attention influences the incorporation of items differing in task relevancy into the spatial configuration, these manipulations should moderate the extent to which changing the locations (between study and test) of the different sets disrupts memory performance.

In the following experiments, participants performed a change-detection task. The initial memory display contained three sets of items (letters, shapes, and objects). Participants were instructed to always ignore one of the sets of items. For each participant, the ‘to-be-ignored set’ remained irrelevant for the entire experiment, but the specific to-be-ignored set was counterbalanced across participants. During the maintenance interval, of the two remaining sets, one was cued as the ‘target set’—the set that the participants should base their decision on and the other set the ‘uncued set’. During the maintenance interval, a verbal cue informed the participants for which set their memory would be tested. The features of the uncued set never changed. One of the items within the target set contained a new feature (colour or shape) on 50% of the trials (*new* feature trials). At test, the task was to indicate whether the target set contained a feature that was not previously present in the memory display. The purpose of this paradigm was to manipulate the task-relevance of different subsets of items—some were always task relevant, while others became task irrelevant during the maintenance interval. In order to test the competing top-down dependent and top-down independent theories, we aimed to measure the extent to which those subsets, which varied in task relevance, formed part of the spatial representation.

In order to measure the extent to which these different sets formed part of the configuration, we used a paradigm employed by Treisman and Zhang (2006). In their paradigm, participants were asked to study displays of coloured shapes, and instructed to ignore the item locations or feature bindings between colour and shape. For example, if a participant initially saw a red square and blue circle, followed by a blue square and a red circle, they should still respond ‘match’, because all four features were originally present, despite having new combinations. Treisman and Zhang found that when the bindings were intact (e.g., when a red square and blue circle remained a red square and blue circle), presenting the probed items at new locations made performance worse than when they had been presented in their original locations. However, when the bindings had been switched, performance was better when the probe items were presented in new locations than when they were presented in their original locations. This interaction between location and binding serves to measure the use of the configuration in retrieval from VWM (Treisman & Zhang, 2006; Udale et al., 2017b). We adapted this paradigm for the present study. In order to measure the effect of task relevance on binding to the spatial configuration, we systematically manipulated whether different subsets of items, which varied in their task relevance, maintained their locations across study and test displays, and whether or not the target set contained its original colour-shape bindings in the probe display.

If participants can control which items are encoded based on the item’s relevance to the task, then the locations of to-be-ignored items (on all trials) should never be encoded, whereas the locations of the remaining two sets (cued and uncued) should be initially encoded, and potentially form the configural representation. The questions addressed by the following series of experiments were (i) whether the locations of to-be-ignored items formed part of the configuration, and (ii) whether items can be removed from the configuration if they subsequently become irrelevant to the task. Changing the location of the three different sets of items between study and test will inform us of whether their position was included in the configural representation. If the position of an item was encoded and maintained, then changing its location should result in lower change-detection performance, relative to when its position had not changed. The main finding from all three experiments was that performance was unaffected by changing the locations of any of the task-relevant or task-irrelevant items in the display. This suggests that, in the context of this particular task, participant can perform change detection without necessarily relying on the spatial configuration of the studied display.

## Experiment 1

If items in a display are automatically bound to the spatial representation, then they should form part of the configuration, irrespective of whether those items are task relevant. Additionally, if the structural gist is unaffected by top-down attention, then it should not be possible to remove locations from a configuration once they have been encoded. To test this, participants were given a change-detection task, containing some items that were never tested, and measured whether those items were bound to the configuration. The locations (and feature bindings of the task-relevant items) of different subsets of items were manipulated in order to measure whether those items formed part of the configuration. This experiment was preregistered on the Open Science Framework (https://osf.io/49fzr/).

### Method

#### Participants

For this experiment, and the others reported here, we recruited 36 naïve participants (ages 18–53 years, 26 females) through the University of Bristol’s Experimental hours’ scheme, and through posters in and around the campus. We selected our sample size based on previous experiments using a similar task showing robust effects (Udale et al., 2017b). Participants were either reimbursed £7 (Approx. $8.56) or took part for course credits. All participants reported fluency in English. All participants reported normal or corrected-to-normal vision. Ethical approval was granted by the Faculty of Science Research Ethics Committee.

#### Materials

The presentation of the stimuli was controlled using MATLAB (The MathWorks, Natick, MA) and the Psychophysics Toolbox (Brainard, 1997; Kleiner, Brainard & Pelli, 2007 2007; Pelli, 1997), using a 17-in. TFT monitor (Resolution: 1,280 × 1,024) with a refresh rate of 60 Hz. Participants’ responses were recorded using a standard USB keyboard. The stimuli were presented on a uniform medium grey background (RGB: 128, 128, 128). The stimuli were conjunctions of nine possible colours, six shapes, six letters, and six objects based on the Snodgrass and Vanderwart (1980) set. Each shape, letter, and object was combined with each colour, resulting in a total of 162 different stimuli, as displayed in Table 1. Each of the study and probe displays consisted of six items, consisting of conjunctions of colours with two from each item set (shapes, letters, or objects). Each item subtended approximately 1.8° × 1.8° visual angle, at a viewing distance of approximately 1 m. Each item could appear in one of nine possible locations. The nine locations formed a 3 × 3 grid, each location consisting of 60 × 60 pixels (1.8° × 1.8° visual angle), with 36 pixels (1.08° visual angle) of empty space between each location. The total grid size was 252 × 252 pixels (7.6° × 7.6° visual angle). The items, colours, and locations were randomly chosen at the start of each trial without replacement.

**Table 1:**
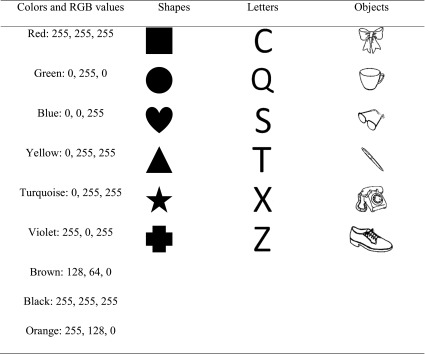
The different colours, shapes, letters, and objects used as stimuli

#### Design

The experimental design was a 2 (target feature: match vs. change) × 2 (target binding: intact vs. switch) × 4 (location: no set change vs. relevant set change vs. ,o-longer-relevant set change vs. irrelevant set change) fully crossed within-subjects design. The first factor was target feature (match vs. change): On half of the trials, all the features of the target set in the memory display were present in the test display (match). On the other half of the trials, one of the features (either colour or shape) of the target set had changed between study and test displays (change). On target feature change trials, one of the two target items was randomly selected, and one of the two features (colour or shape) of the selected item was randomly selected to be replaced with a feature that was not present in the study display. For example, if a red square and blue circle were studied, a yellow square and blue circle might be presented at test. The second factor was finding (intact vs. switched). On half of the trials. the feature bindings of the target set switched (e.g., a red square and blue circle in the study display would subsequently become a red circle and a blue square). Colour and shape switches occurred equally often. On the other half of trials, binding switches did not occur, so that the items maintained their original feature bindings. The third factor was location change. This factor systematically varied which of the stimuli sets (no change, relevant change, no-longer-relevant change, or irrelevant change) changed locations between the study and test displays. In the no-change condition, all test items appeared in the locations they occupied during the memory display. In the relevant change condition, the targets appeared in a location that they had not occupied in the memory display. In the no-longer-relevant change condition, the no-longer-relevant items (i.e., items that were not cued in the maintenance interval, but were cued on other trials) were presented in new locations in the test display that they had not occupied in the memory display. Finally, in the irrelevant change condition, the irrelevant item set (those which were never tested at any point during the experiment) were presented in locations in the test display that they had not occupied in the memory display. If any one of the sets changed locations on a trial, all of the other sets remained in their original locations. The visual features of the irrelevant set remained the same throughout the whole experiment, and the type of item (letter, shape, or object) was counterbalanced across participants.

#### Procedure

At the start of the experiment, participants were informed about the nature of the working memory task. The participants were informed that they could always ignore one of the three stimulus sets in the memory display and were told which set this was. They were also told that their memory would only ever be tested for one of the two other stimulus sets. Participants were informed that during the maintenance period of each trial they would be given an instruction about which stimulus set would subsequently be probed. Finally, participants were instructed not to base their responses on the locations of the stimuli, or their feature bindings.

**Fig. 1:**
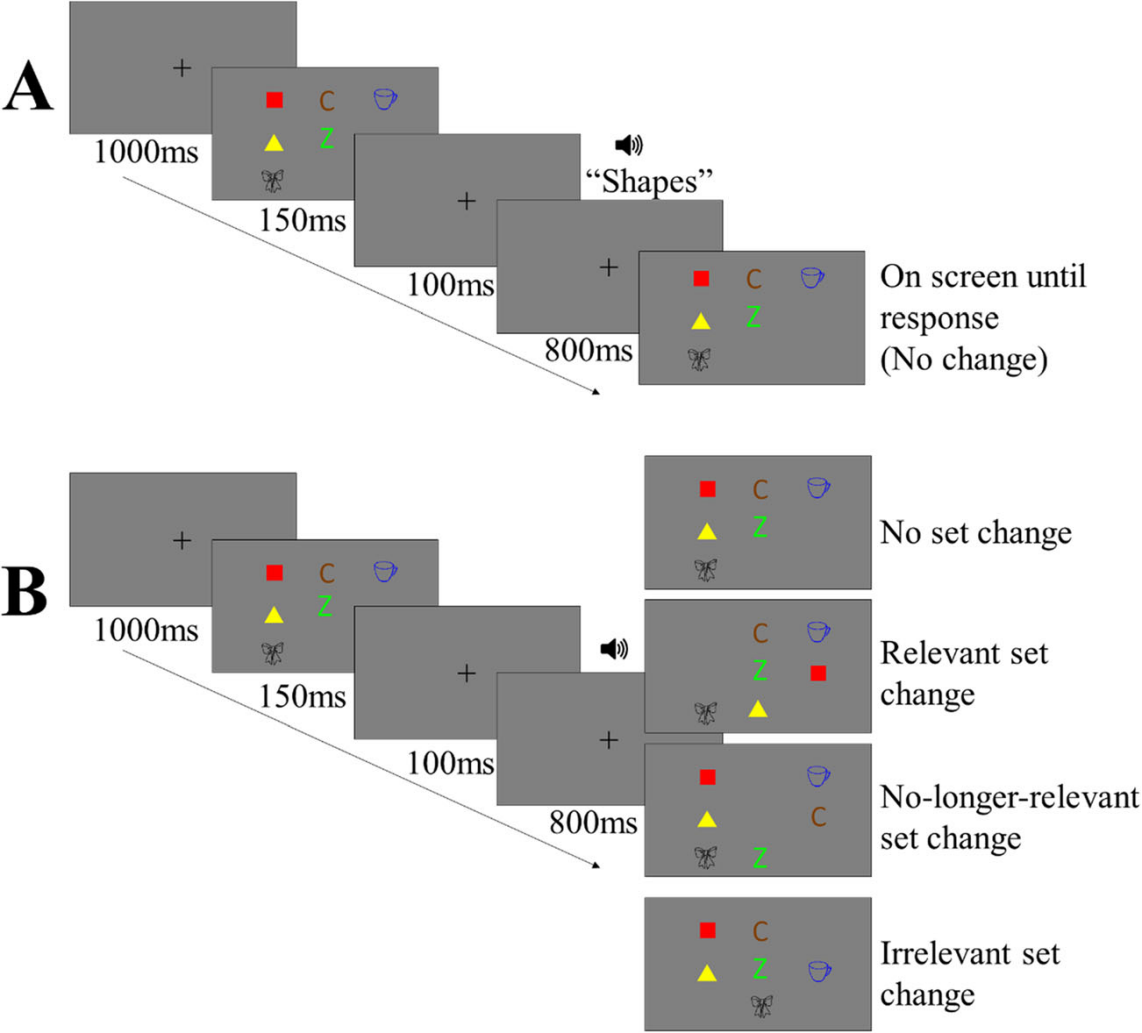
**a** Schematic of the procedure and timings used in Experiments 1–3. **b** Examples of the different location change conditions. In both schematics, objects are the always irrelevant set, shapes are the target set, and letters and the no-longer-relevant set. (Colour figure online)

On each trial, a small black fixation cross appeared at the centre of the screen for 1,000 ms, followed by a study display for 150 ms, which contained two randomly selected without replacement items from each of the three stimuli classes. The study display was followed by a blank maintenance screen, with a centrally presented fixation cross for 900 ms. During the maintenance interval, 100 ms after the offset of the memory display, a prerecorded voice was played, stating either ‘Letters’, ‘Shapes’, or ‘Objects’, which informed the participant which stimuli set was the target set (i.e., for which of the sets their memory would subsequently be probed). After the maintenance interval, a test display was presented containing the three pairs of items. Participants were asked to indicate whether a new feature was present amongst the target set. Participants were instructed to ignore any changes in locations or combinations of feature bindings, and only to base their decision on whether or not a new feature was present amongst the target set. The test display remained on-screen until a response was made. The participants responded using the *F* and *J* keys, with mappings counterbalanced across participants. Participants were also asked to favour responding accurately over responding quickly. They were also asked to perform articulatory suppression by repeating ‘Coca-Cola’ throughout each trial at the rate of about once per second in order to inhibit verbal recoding. A schematic of the procedure and the different location change conditions are presented in Fig. 1.

The testing session consisted of three practice blocks, followed by four experimental blocks. The purpose of giving the participants three practice blocks was due to the difficult nature of the task. Each practice block, consisting of 16 trials each, was designed to be successively more difficult, until it matched the task in the main experiment. In the first practice block, only two sets were presented (always attend and always ignore), and items maintained their location between study and test. In the second block, changes in their locations were introduced. Finally, all three item sets were used in the third practice block, matching the full experiment. The experiment consisted of four blocks, with 128 trials in each block, resulting in a total of 512 trials per participant. Per block, there were 64 match trials, and 64 change trials. These match and change trials were crossed with 64 trials of each binding condition. This was crossed with 32 trials of each location change condition per block. In total, there were 16 possible conditions, from all possible combinations of all the levels of the three factors. This meant that within each block, every condition was repeated eight times, and each condition was repeated 32 times throughout the entire experiment for each participant. The order of the trials was randomly intermixed.

#### Statistical analysis

So that we only included participants in the analysis who were engaging with the task (i.e., performing above chance), we excluded participants from the analysis if their corrected hit rate (hit rate minus false alarm) fell below 0.1 in the ‘intact binding, match feature, old location’ condition. This condition was chosen to assess inclusion for two reasons. First, we did not want to exclude participants based on grand average performance, because some conditions, such as when the bindings have switched, may pull this average down. Secondly, we chose this condition because it was considered to be the easiest condition, and therefore should be the most inclusive criteria.

Although we report our conclusions based on null hypothesis significance testing, we have also supplemented our analyses using a Bayesian analysis of variance. The Bayesian approach provides the advantage that it allows one to specify and competitively test null and alternative hypotheses. In contrast to the frequentist approach, where inferences about differences are made on the lack of evidence for a null hypothesis, Bayesian methods provide the relative evidence in favour of the null or alternative hypotheses. Because Bayes factors represent relative evidence between the two hypotheses, indices (BF_10_ for the alternate and BF_01_ for the null) are used to indicate which hypothesis the Bayes factor is describing. For example, a BF_10_ of 9 indicates that there is nine times more evidence for the alternate than for the null hypothesis. Because this value is a ratio, it can also be represented as the amount of evidence for the null: A BF_10_ of 10 is equivalent to a BF_01_ of 0.1. We conducted our analysis using the anovaBF function from the BayesFactor package (Rouder, Morey, Speckman, & Province, 2012) in R (R Core Team, 2017), using the default JZS prior settings in all analyses (cf. Bayarri & García-Donato, 2007; Jeffreys, 1961; Rouder et al., 2012; Zellner & Siow, 1980), as we find these to be plausible priors for the domain of VWM.

### Results

**Fig. 2:**
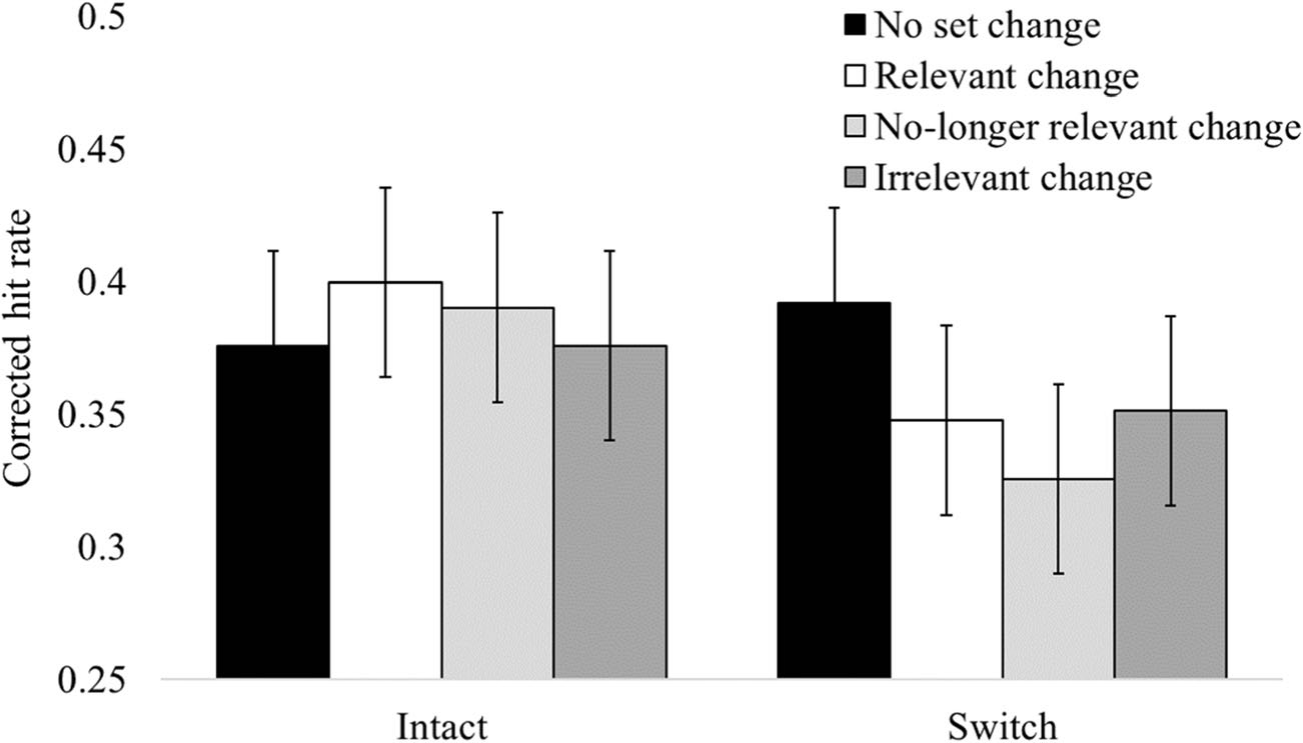
Mean corrected hit rate for each condition in the location and binding factors. Error bars represent 95% within-subjects confidence intervals using the Loftus and Masson (1994) calculation

Using the exclusion criteria described above, the data from three participants were excluded from the analysis, leaving a total of 33 participants for analysis. Furthermore, we removed individual trials if the response time was greater than 4 seconds, or less than 100 ms. As a result, a total of 384 trials (2.27%) were removed (*M* = 11.63, *SD* = 18.41 per participant). Figure 2 plots the mean corrected hit rates for each condition. A 4 (location: no set change vs. relevant set change vs. no-longer-relevant set change vs. irrelevant set change) × 2 (binding: intact vs. switch) repeated-measures ANOVA was conducted on the corrected hit rates. There was no main effect of location, *F*(3, 96) = 0.61, *p* = .609, BF_01_ = 26.01, but there was a significant effect of binding, *F*(1, 32) = 4.71, *p* = .037, ηp^2^ = 0.13, BF_10_ = 1.49, in which average performance was lower in the binding switched condition (.35) than in the binding intact condition (.38). The Location × Binding interaction was also not significant, *F*(3, 96) = 2.04, *p* = .114, BF_01_ = 2.86.

Finally, we performed a 2 (location: no set change vs. relevant set change) × 2 (binding: intact vs. switched) ANOVA on corrected hit rates, in order to more closely replicate the analysis performed in previous studies (Treisman & Zhang, 2006; Udale et al., 2017a, b), which found a detrimental effect of location changes when bindings were intact, but a beneficial effect of location changes when bindings were switched, as well as an overall detrimental effect of switching bindings. We found no main effect of location, *F*(1, 32) = 1.08, *p* = .307, BF_01_ = 2.97, or binding, *F*(1, 32) = 0.06, *p* = .805, BF_01_ = 5.17, and the two-way interaction was not significant, *F*(1, 32) = 1.28, *p* = .266, BF_01_ = 2.56.

### Discussion

Experiment 1 found no evidence that changing the location of any of the items in the display had any effect on performance, when compared with the condition in which all items remained in their original locations. This suggests that participants did not make use of item locations to guide the comparison process for the target items. This interpretation was further bolstered by the finding that, when directly replicating the analysis of previous studies (Treisman & Zhang, 2006; Udale et al., 2017a, b), we did not find an effect of location, binding, or a two-way interaction between location and binding. This runs contrary to the findings of previous experiments, which have found that changing the locations of at least target items disrupts memory performance (Boduroglu & Shah, 2006; Hollingworth, 2006; Jiang et al., 2000; Treisman & Zhang, 2006; Udale et al., 2017a, b), and might suggest that location is not obligatorily reinstated during the response stage of the task. It is unclear why changing the locations of the targets had no effect on performance; however, we speculate on some possible explanations in the general discussion.

## Experiment 2

Experiment 2 aimed to address one of the potential methodological issues with Experiment 1. Specifically, although participants knew ahead of each trial which sets were task relevant or irrelevant, they still needed to attend to each item in the study display in order to perceive it, and assign it to its set, before deciding whether or not that particular item was relevant. Simply making this relevant-or-not decision may have led participants to encode the task-irrelevant items (cf. Olson et al., 2008). Therefore, in order to test this possible account of the null effects in Experiment 1, we replicated Experiment 1, but provided spatial cues prior to the onset of the study display so that participants knew ahead of time where the task-relevant items were going to appear. This gave the participants the opportunity to encode only the locations of the task-relevant items, because they would not need to attend to, or make any decisions about, the task-irrelevant items. Additionally, we manipulated the validity of the cue—either two cues appeared in the locations of the target items—so that participants only needed to encode the set which would subsequently be probed, or four cues appeared, so that both the targets and no-longer-relevant items (the set that became irrelevant during the maintenance period) were also cued. A cue-absent condition, in which no precues were presented, was also included as a replication of Experiment 1.

### Method

#### Participants

We recruited 36 new participants (ages 18–46 years, 25 females).

#### Stimuli, design, and procedure

**Fig. 3:**
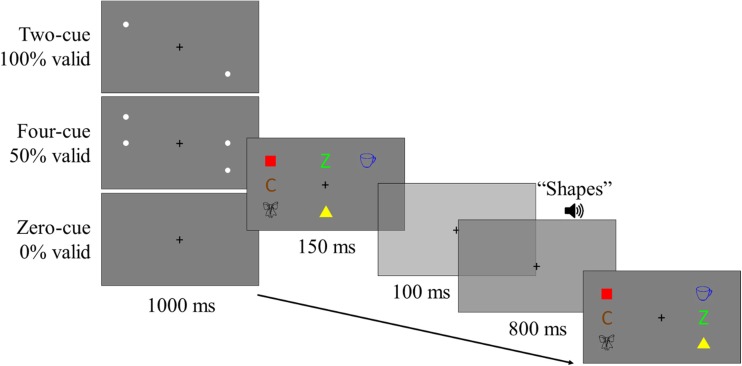
Schematic of a typical trial in Experiment 2, with the three possible precue conditions. (Colour figure online)

The stimuli, design, and procedure were identical to that of Experiment 1, except for an additional factor (cue). At the start of each trial, precues appeared prior to the study display, which indicated the locations where the to-be-remembered items would subsequently appear in the study display. The cues appeared for 1,000 ms and offset at the same time as the onset of the study display. On one third of trials, no cues appeared (zero cue), such that participants would need to encode all of the displayed items, irrespective of their task relevance. On another third of trials, four cues appeared in the locations of all four to-be-remembered items (four cue), such that they did not need to encode the always irrelevant set, which was never probed. On a final third of trials, only two cues appeared in the locations of the items which would subsequently become the target set in the test display (two cue), such that they only needed to encode the two items which would subsequently be probed. We maintained the number of trials in each cell of the design, and with the addition of this three-level factor, the experiment now ran for 3 hours, over three sessions on different days. A schematic of a typical trial from Experiment 2 is presented in Fig. 3.

### Results

**Fig. 4:**
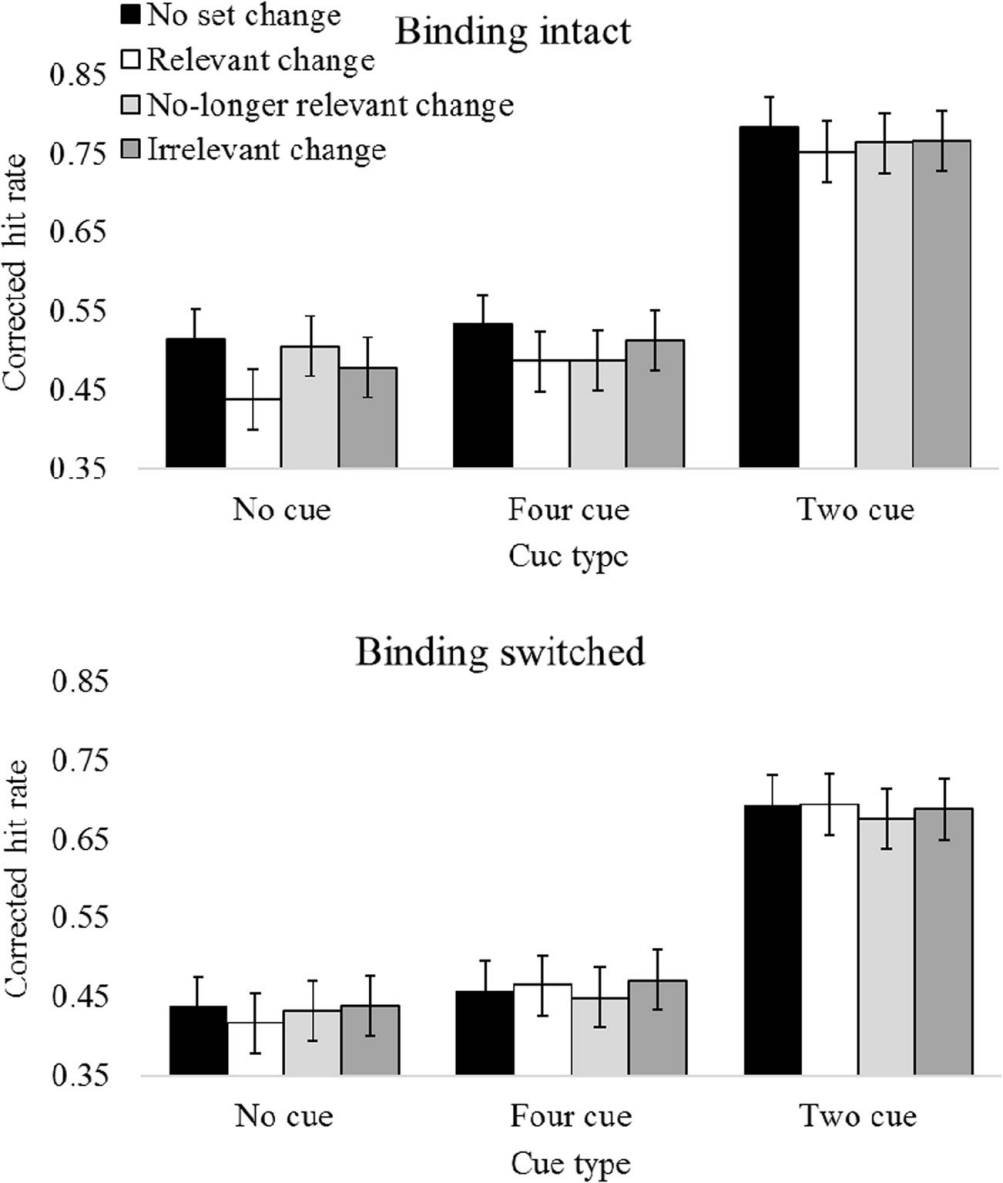
Average corrected hit rates for each condition in the cue, location, and binding factors of Experiment 2. Top: Intact binding trials. Bottom: Switched binding trials. Error bars represent 95% within-subjects confidence intervals using the Loftus and Masson (1994) calculation

Using the same exclusion criteria as in Experiment 1, zero participants were removed, and a total of 668 (3.62%) trials were removed (*M* = 18.55, *SD* = 41.13, per participant). Figure 4 represents the average corrected hit rates for each condition in Experiment 2. A 3 (cue: zero cue, two cue, four cue) × 4 (location: no change vs. relevant change vs. no-longer-relevant change vs. irrelevant change) × 2 (binding: intact, switched) repeated-measures ANOVA was conducted on the corrected hit rates. There was a main effect of cue, *F*(2, 70) = 70.61, *p* < .001, ηp^2^= 0.67, BF_10_ = 4.5 × 10^109^, with performance higher in the two-cue condition (.73) than in the four-cue (.48), or zero-cue (.46) conditions. There was also a significant effect of location, in which changing the location of any set appeared to marginally reduce performance, relative to the condition in which no items changed locations, *F*(3, 105) = 2.9, *p* = .038, ηp^2^= 0.07, BF_01_ = 51.19. However, any conclusions about this effect should be drawn with caution, when taking into account the very large Bayes factor in favour of the null hypothesis. There was also a significant effect of binding, *F*(1, 35) = 46.47, *p* < .001, ηp^2^= 0.57, BF_10_ = 5069.09, in which performance was worse when the bindings had switched (.53) than when they were intact (.59), which was also supported by a strong Bayes factor in support of the alternative hypothesis. None of the interactions were significant: Cue × Location, *F*(6, 210) = 1.24, *p* = .286, BF_01_ = 450.84; Cue × Binding, *F*(2, 70) = 1.82, *p* = .169, BF_01_ = 20.3; Location × Binding, *F*(3, 105) = 2.02, *p* = .116, BF_01_ = 44.25; and Cue × Location × Binding, *F*(6, 210) = 0.23, *p* = .966, BF_01_ = 183.45.

### Discussion

Experiment 2 replicated the findings from Experiment 1: Changing the locations of any of the sets had little effect on performance, when compared with the condition in which none of the items changed locations. There was a significant effect of location. However, this should be interpreted with caution, due to the small effect size (ηp^2^= 0.07), and the fact that the corresponding Bayes factor was in strong support of the null hypothesis (BF_01_ = 51.18). Furthermore, the Location × Binding and Cue × Location × Binding interactions were not significant, further suggesting that participants did not bind the different sets to the configuration, despite the provision of cues that encouraged configuration binding of the cued items, but not the uncued items. The finding that participants performed better in the two-cue condition than in either of the other cue conditions suggests that participants were utilising the cue, so we can assume that they were able to distinguish between relevant and irrelevant items. As with Experiment 1, these results further support a view of VWM in which the locations of individual items are not necessarily bound to the structural gist of the display, or that the structural gist is not necessarily reinstated during the decision stage of the change-detection task.

## Experiment 3

Experiment 3 was conducted in order to overcome a potential methodological problem with Experiments 1 and 2. Specifically, in the two previous experiments, the locations of the studied items were randomly intermixed on a 3 × 3 grid. This could lead to situations where items from different sets were spatially intermixed; for example, a to-be-ignored irrelevant item might appear in a location between two to-be-remembered target items. With this design, it might have been necessary to shift attention across the display, between the relevant items. If to-be-ignored items were presented between two to-be-remembered items, the shift between the relevant items might incidentally incorporate the interposed irrelevant item. As a result, it was still possible that participants attended to the irrelevant items whilst encoding the target items. To better control for this potential issue, we replicated Experiment 2, but presented each of the stimulus sets in separate quadrants of the display. When one of the item sets changed locations in the test display, it would only change locations within its own quadrant. This design choice should reduce the chances of participants attending to, and encoding, the to-be-ignored items. Finally, because the binding manipulation had not been very diagnostic in the previous two experiments, to reduce the complexity of the design and increase the number of observations per cell, we removed the switched binding condition from the task altogether.

### Method

#### Participants

We recruited 36 new participants (ages 17–25 years, 32 females).

#### Stimuli, design, and procedure

**Fig. 5:**
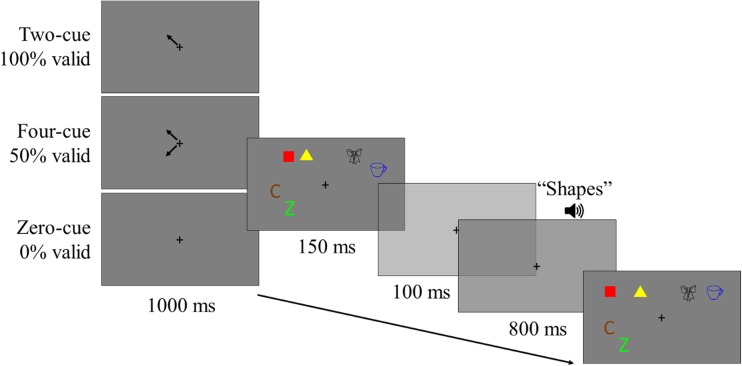
Schematic of an example trial in Experiment 3. In this example, shapes are the target set, which have been presented in a new location within their quadrant. This is an example of a new feature condition. (Colour figure online)

For Experiment 3, the following changes were made to the method of Experiment 2. First, the three pairs of items were presented in separate quadrants of the display, so that a quadrant only contained the items from within the same set. Additionally, when item locations changed, they changed to a new location within their quadrant. Each quadrant contained four possible locations, composed of a 2 × 2 grid. Each item was presented at 60 × 60 pixels (as with the previous experiments), with three pixels between adjacent items within the same quadrant. Thus, each quadrant was 123 × 123 pixels. There were 65 pixels between the edges of adjacent quadrants. Thus, the total display consisted of 311 × 311 pixels. Second, the peripheral precues were replaced with centrally presented arrow precues, which pointed to the quadrant where the to-be-remembered items would subsequently appear. The purpose for this change in design was to further increase the salience and predictability of the cue, and therefore to increase the probability that the participants only attended to the to-be-encoded items. A single arrow was presented in the 100% validity condition, whereas two arrows were presented in the 50% validity condition. It should be pointed out here that the condition labels of ‘two cue’ and ‘four cue’ refer specifically to the number of items which are being cued, rather than the number of cues, in order to maintain consistency in the labels. Finally, the binding switch trials were removed, so that all conditions were ‘intact’ binding trials. As a result, the number of trials in the other conditions was doubled, so that there were 512 trials in total. The schematic of an example trial is presented in Fig. 5.

### Results

**Fig. 6:**
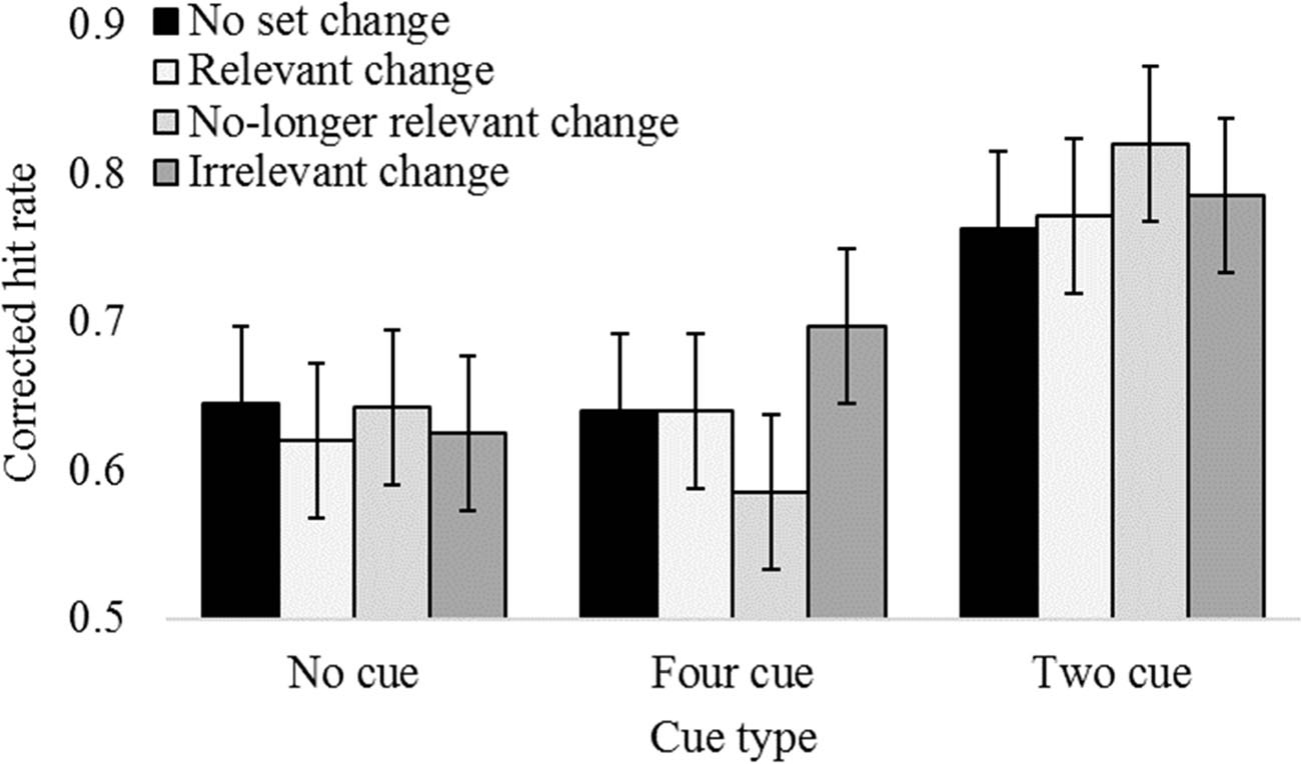
Average corrected hit rates for each condition in the cue and location factors of Experiment 3. Error bars represent 95% within-subjects confidence intervals using the Loftus and Masson (1994) calculation

Using the same exclusion criteria as in the previous experiments, all participants were included in the analysis, and a total of 92 (0.51%) trials were removed (*M* = 2.62, *SD* = 6.56, per participants). Figure 6 reports the average corrected hit rate for each condition in Experiment 3. A 3 (cue: no cue, two cue, four cue) × 4 (location: no change vs. relevant change vs. no-longer-relevant change vs. irrelevant change) repeated-measures ANOVA was conducted on the corrected hit rate data. There was a significant main effect of cue, *F*(2, 68) = 16.55, *p* < .001, ηp^2^ = 0.33, BF_10_ = 3.8 × 10^11^, in which participants performed better in the two-cue condition (.79) than in the four-cue (.64) or zero-cue (.63) condition. The main effect of location was not statistically significant, with the Bayes factors strongly favouring the null hypothesis, *F*(3, 102) = 0.63, *p* = .6, BF_01_ = 62.24. The Cue × Location interaction was also not statistically significant, *F*(6, 204) = 1.89, *p* = .085, BF_01_ = 14.72.

### Discussion

Experiment 3 further replicates the findings of Experiments 1 and 2: Changing the locations of any of the items in the display, including the target items, had no effect on performance, relative to the condition in which no items changed locations between memory and probe displays. Likewise, there was no moderating effect of the cue factor on the effect of location. As with Experiment 2, participants performed better in the two-cue (100% valid) condition than in either of the other cue conditions, suggesting that they were able to utilise the cue, and therefore we can be relatively confident that they were able to distinguish between relevant and irrelevant items. These findings further support a view of VWM in which the structural gist is not necessarily reinstated during the decision stage of the change-detection task.

## General discussion

Across all three experiments, we did not find any convincing evidence that participants were binding the display items to their relative positions in the spatial configuration. Furthermore, the null main effects of location and null Location × Binding interactions were supported by Bayes factors in favour of the null hypothesis. For Experiment 1, the Bayes factors for the null are in the ‘ambiguous’ evidence range for the effect of location (BF_01_= 2.96) and Location × Binding interaction (BF_01_ = 2.86). However, in Experiments 2 and 3, the Bayes factors are quite strongly in favour of the null (all BFs_01_ > 14.72). Furthermore, in Experiment 2, where there was a significant effect of location according to the frequentist analysis, the Bayes factor still showed strong preference against an effect of location. Taken together, although conclusions should be drawn cautiously about the absence of an effect on the basis of Experiment 1, we can be more confident about the lack of an effect based on Experiments 2 and 3. It was surprising that there appeared to be very little effect of the location manipulation, considering the large body of literature that finds poorer performance on trials when the probed items have been presented in new locations compared with their old locations. We expected to find that at least changing the locations of the target set would disrupt performance; however, even this was not the case. These findings are inconsistent with a large body of literature suggesting that participants automatically, or obligatorily, reinstate the initial studied configuration in order to make a change-detection response (Boduroglu & Shah, 2006; Golomb, Kupitz, & Thiemann, 2014; Hayes, Nadel, & Ryan, 2007; Hollingworth, 2006, 2007; Jiang et al., 2000; Kondo & Saiki, 2012; Mou et al., 2008; Olivers & Schreij, 2011; Papenmeier, Huff, & Schwan, 2012; Silvis & Shapiro, 2014; Treisman & Zhang, 2006).

One possible account for the data here is that the experiments were quite complicated, and so participants did not understand what their task was, and therefore did not encode the studied items in the first place, which may have led to them not representing the structural gist. However, the fact that performance is well above chance suggests that the participants did indeed understand the task instructions, and therefore we should expect that the remembered visual features would be bound to the structural gist representation. Additionally, the fact that there is a very strong benefit in the two-cue (100% valid) conditions of Experiments 2 and 3 suggests that the participants were engaging with the task and understood how they could make use of the cue.

An alternative explanation for the lack of any effects of location change in these experiments relies on a strategic account of how participants approached the task (Udale et al., 2017b). It is possible that participants encode the structural gist, but for strategic reasons did not make use of it in the retrieval and decision stage of the task. One strategic reason for not using the structural gist in these experiments is that item locations were less diagnostic in these experiments than in previous studies that have manipulated item locations. Specifically, 75% of trials contained some location change (relevant change, no-longer-relevant change, irrelevant change), and only 25% of trials contained the same spatial configuration that was initially studied (no change). In contrast, previous studies that find effects of location change have done so with a 50% probability of a location change on each trial. Reinstating the global configuration during the decision stage may not have been a beneficial strategy, because at least part of the spatial configuration changed on 75% of the trials, and therefore possibly discouraged the participants from making use of the configuration.

Furthermore, the probe displays may be more akin to ‘partial probes’, in which the participants only needed to make a decision about a subset of the display. Specifically, after a few hundred milliseconds from the onset of the maintenance interval, the participants knew that they would only be deciding about two items (after hearing the verbal cue), and that they could ignore the other four items presented in the test display. In contrast, many studies providing evidence for spatial configurations in VWM have utilised whole-display probes, in which all of the probed items are task relevant (Hollingworth, 2006; Jiang et al., 2000; Treisman & Zhang, 2006). Because the decision only needs to be made about a subset of items in the display, rather than all of the probed items, participants may be less likely to rely on the structural gist. For example, relying on the structural gist might involve parallel old/new decisions about each item in the display (cf. Wilken & Ma, 2004). Since it is unnecessary to make a decision about every item in the display, the participants might rely on some other (nonspatial) comparison strategy, which does not rely on the structural gist (Udale et al., 2017b). Support for this explanation comes from Papenmeier et al. (2012), who had participants view multiple item memory arrays, and tested their memory using either ‘whole’ probes, in which all of the to-be-remembered items were presented again at test, or ‘partial’ probes, in which only a subset of the studied items were presented again at test. When the orientation of the display changed, such that the locations of the probed items no longer matched the locations of the study items, performance dropped more in the whole-probe condition than in the partial-probe condition. To summarise, participants may only rely on the structural gist in tasks which rely on making task-relevant decisions about all of the items in the display, rather than when only a subset of the presented items are task relevant, or when only a subset of the studied items are presented again at test.

We propose a third account for the lack of location effects in our experiments, as well as those relying on partial probes in previous studies (Papenmeier et al., 2012). Specifically, there is a difference in decision load between the typical whole-probe design and ours (as well as partial probes), in which old/new decisions are made about a subset of the display. When participants must make a change-detection decision about the entire display, they may benefit more from utilising the structural gist in order to reduce the number of comparisons. However, because partial probes have a lower decision load than whole probes, there may be a smaller benefit conferred from utilising a location-based comparison process, and instead participants might conduct a nonspatial search of memory (Udale et al., 2017b). Previously, we have proposed that participants may use different comparison strategies depending upon the specific task demands (Udale et al., 2017b). For example, when presented with a whole probe, in which a change might occur in any item, it may be strategically beneficial to conduct ‘global in-place matching’, whereby each probed item is compared with the item at the corresponding location in the study display (and no others). This comparison strategy is similar to the *maximum absolute difference* model (Wilken, 2001; Wilken & Ma, 2004) or the *independent decision* model (Shaw, 1980; Palmer, 1990). However, when tasked with a decision about a subset of items, there is no need to conduct exhaustive in-place matching, but instead participants can conduct a nonspatial search for those items, in which the probed item/s are compared with multiple items in memory. This idea is supported by the finding that when presented with a single probe, whereby only a single item is presented in the probe display, performance is unaffected by irrelevant location changes (Gilchrist & Cowan, 2014; Treisman & Zhang, 2006; Udale et al., 2017a). Further, conducting either in-place matching or nonspatial search seems to be subject to task demands. For example, when presented with a whole-display probe, changes in the locations of the configuration disrupt memory performance (Hollingworth, 2006, 2007; Jiang et al., 2000; Sun & Gordon, 2010), suggesting that participants conduct in-place matching. However, when asked to make a decision about a single item within the display (identified with a cue), participants’ performance is unaffected by configuration changes (Udale et al., 2017a), implying that they conducted a nonspatial search for the target item. In the present experiments, participants were asked to make a decision about only two items within a six-item display. The decision load for the two items here may be larger than a single probe, but may still have been small enough to permit a strategy other than in-place matching, and one that is not sensitive to location changes, as we see for single probe displays.

What are the theoretical implications of our experiments? The primary research question we addressed was whether or not the locations of task-irrelevant items necessarily enter the configuration representation in VWM. Some accounts of VWM hold the assumption that all item locations are automatically bound to the configuration representation (‘structural gist’), and that this is obligatorily reinstated during retrieval. We have provided evidence across three experiments that this does not necessarily occur, and further still, the locations of highly task-relevant items may not be bound to the structural gist, or if they are bound, then this information is not utilised in the retrieval and decision stages. Thus, maintaining feature location binding and reinstatement of the original configuration is not necessary for accurate change detection. We propose an alternative account, in which the extent to which participants encode and retrieve the studied configuration depends on the decision load of the task, and that they will not utilise the configuration if they only need to detect changes in a small number of items, rather than the entire display. One caveat to these experiments is that, because we could not show a condition in which presenting items in their original locations was more beneficial than changing the locations, we cannot draw strong conclusions about why they did not utilise the spatial configuration. Further research is therefore needed in order to provide an explanation for why location is not utilised in partial-probe designs, and how participants can perform change detection accurately without utilising location to guide the comparison process.

### Author note

This research was funded by a studentship in teaching and research from the Faculty of Science at the University of Bristol. The second author was supported by an Australian Future Fellowship (FT130100149).

We would like to thank Hugo Hammond, Kira Griffiths, Maria Antoniou, Shanaz Pottinger, Veronika Hadjipanayi, and Yuxuan Bo for their help with data collection.
